# Biological Characteristics, Nutritional Composition, and Heavy Metal Safety of Mycelia from *Gerhardtia borealis*, a Rare Wild Edible Fungus

**DOI:** 10.3390/life16060967

**Published:** 2026-06-08

**Authors:** Yufan Jin, Di Zhang, Yusen Liu, Yunjiang Liang, Jize Xu

**Affiliations:** 1 Agricultural College, Yanbian University, Yanbian 136200, China; 2 Agricultural College, Jilin Agricultural Science and Technology University, Jilin City 132101, China

**Keywords:** *Gerhardtia borealis*, biological characteristics, nutritional components, orthogonal test, ITS identification

## Abstract

The orthogonal design further optimized the culture medium to a combination of ammonium nitrate, sucrose, and magnesium sulfate, achieving a mycelial growth rate of 1.379 mm/d. The mycelia of *Gerhardtia borealis* contained 26.01% crude protein, 6.03% crude fat, and 1.24% crude polysaccharides. A total of 17 amino acids were detected, with a total content of 26.09 g/kg. The iron and zinc contents in the mycelia were 28.09 mg/kg and 22.17 mg/kg, respectively. The concentrations of arsenic, cadmium, mercury, and lead were all below the national food safety limits. This study provides fundamental data supporting the domestication and functional utilization of *Gerhardtia borealis* as an edible and medicinal resource.

## 1. Introduction

*Gerhardtia borealis* (Fr.) Contu & A.Ortega is a fungal species belonging to the phylum Basidiomycota, class Agaricomycetes, order Agaricales, and family Lyophyllaceae, within the genus *Gerhardtia*. The genus *Gerhardtia* was initially regarded as a subgenus of *Lyophyllum* but was later elevated to generic rank [1]. In 1994, Bon provided a comprehensive morphological description of *G. borealis* using traditional methods, marking its formal establishment as an independent taxonomic unit. The main characteristics of this genus include a variably colored pileus, a pileipellis that may be a cutis, trichoderm, or hymeniderm, and basidiospores that appear smooth or slightly undulate under light microscopy. Although several *Gerhardtia* species distributed in Europe and the Americas have been morphologically described, research on *G. borealis* in China remains scarce to date, with no detailed reports on its biological characteristics, nutritional composition, or artificial domestication potential.

Edible and medicinal fungi have garnered global attention not only for their culinary value but also for their significant nutritional and pharmacological properties. World-renowned commercial species such as *Agaricus bisporus* (white button mushroom), *Pleurotus ostreatus* (oyster mushroom), *Lentinula edodes* (shiitake), and *Morchella* spp. (morels) are cultivated on a large scale and have been extensively studied for their high-quality protein, dietary fiber, and bioactive compounds [2]. Similarly, ectomycorrhizal fungi like *Tuber* spp. (truffles) and *Boletus edulis* (porcini) command premium prices due to their unique flavors and aromas, with their nutritional and economic value being well documented [3]. The family Lyophyllaceae, to which *G. borealis* belongs, also comprises a group of edible and medicinal fungi with recognized nutritional properties. Species within this family, such as *Lyophyllum cinerascens* and *Leucocybe connata*, are characterized by high crude protein content (32–33%), low fat content (<2%), and the presence of bioactive compounds including polysaccharides, organic acids, and flavonoids [4,5]. For instance, the crude protein content in fruiting bodies of L. cinerascens reaches 33.46%, with essential amino acids accounting for 40.2% of total amino acids [4]. *Lyophyllum decastes* is also rich in beneficial minerals, containing 50.0 mg/kg zinc, 19.0 mg/kg copper, and 0.013 mg/kg selenium [6]. These nutritional attributes align well with modern dietary requirements for high-protein, low-fat, and functional foods.

Despite the well-documented nutritional and economic significance of many edible fungi worldwide, *G. borealis* remains a largely unexplored species. Unlike commercially established species such as *A. bisporus*, *L. edodes*, or *Pleurotus* spp., which benefit from decades of domestication and cultivation research, no studies have yet reported the biological characteristics, nutritional composition, or artificial domestication potential of *G. borealis*. This knowledge gap hinders the evaluation of its potential as a novel edible and medicinal resource. To address this, the present study focused on a wild strain of *G. borealis* collected from the Qinling Mountains in Gansu Province, China, which was identified through morphological characterization and ITS sequence analysis. The objectives were to (1) investigate the optimal culture conditions for mycelial growth using single-factor and orthogonal experimental designs, (2) determine the basic nutritional composition, amino acid profile, and trace element contents of the mycelia, and (3) evaluate the heavy metal safety of the mycelia in accordance with national food safety standards. The results are expected to provide a theoretical basis and data support for the artificial domestication, cultivation, and germplasm innovation of this species, thereby contributing to the diversification of edible and medicinal fungal resources.

## 2. Materials and Methods

### 2.1. Samples

The specimen of *G*. *borealis* was collected from the Qinling Mountains, Gansu Province, China. A voucher photograph was deposited under the number HMJU10471. The corresponding strain was isolated from the fruiting body.

### 2.2. Morphological and ITS-Based Identification

Macromorphological characteristics of *G*. *borealis*, including lamellae, pileus, stipe, size, color, and texture, were recorded. Identification was performed with reference to the *Atlas of Large Scale Fungal Resources in China* [7].

Genomic DNA was extracted using the Ezup Column Fungi Genomic DNA Purification Kit according to the manufacturer’s instructions. The internal transcribed spacer (ITS) region was amplified using primers ITS1F (5′-CTTGGTCATTTAGAGGAAGTAA-3′) and ITS4 (5′-TCCTCCGCTTATTGATATGC-3′) [8]. PCR amplification was performed in a 25 µL reaction mixture containing 10 µL 2 × M5 HiPer plus Taq HiFi PCR Mix,7 µL ddH_2_O, 7 µL genomic DNA, 0.5 µL forward primer, and 0.5 µL reverse primer. The amplification program consisted of an initial denaturation at 94 °C for 5 min, followed by 30 cycles of denaturation at 94 °C for 30 s, annealing at 53 °C for 30 s, and extension at 72 °C for 30 s, with a final extension at 72 °C for 5 min [9]. PCR products were analyzed by 1% agarose gel electrophoresis using a JY 600 electrophoresis system (Beijing Junyi Electrophoresis Co., Ltd., Beijing, China) and subsequently sequenced by BGI Co., Ltd. (Beijing, China).

### 2.3. Biological Characteristics Study

#### 2.3.1. Effect of pH on Mycelial Growth

The pH of the modified PDA medium was adjusted to five levels (pH 5.0, 6.0, 7.0, 8.0, and 9.0) using 1 mol/L HCI and 1 mol/L NaOH. The medium composition was as follows: potato 200 g, glucose 20 g, potassium dihydrogen phosphate 2 g, magnesium sulfate 1 g, agar 20 g, and distilled water to 1000 mL [10]. After sterilization and cooling, the medium was poured and inoculated under aseptic conditions in a laminar flow hood. Mycelial plugs (0.5 cm in diameter) were excised from actively growing cultures using a sterile cork borer and placed at the center of each plate. The plates were incubated at 25 °C. Each treatment included five replicates. Mycelial morphology, including colony color and growth vigor, was recorded at regular intervals. After 7 days of inoculation, colony diameters were measured using the cross-measurement method, and the average daily growth rate was calculated as follows: Hyphal growth rate (mm/d) = [colony diameter (mm)−fungal block diameter (mm)]/number of growth days (d). In this unit, “d” stands for “day” [11]. Statistical analysis was performed using Duncan’s multiple range test in SPSS version 26.0 to determine significant differences among treatments.

#### 2.3.2. Effect of Carbon Sources on Mycelial Growth

Glucose in the comprehensive PDA medium was individually replaced with sucrose, fructose, starch, or lactose as the sole carbon source. A carbon-free medium was used as the blank control. Each treatment was performed in five replicates. Experimental procedures and analytical methods were consistent with those described in Section 2.3.1.

#### 2.3.3. Effect of Nitrogen Sources on Mycelial Growth

Five nitrogen sources, namely beef extract, peptone, ammonium nitrate, urea, and potassium nitrate, were incorporated into the comprehensive PDA medium. A nitrogen-free medium served as the blank control. Each treatment included five replicates. Experimental procedures and data analysis were conducted as described in Section 2.3.1.

#### 2.3.4. Effect of Inorganic Salts on Mycelial Growth

Five inorganic salts, namely magnesium sulfate, ammonium sulfate, potassium chloride, sodium chloride, and ferric sulfate, were added to the comprehensive PDA medium. A medium without inorganic salts was used as the blank control. Each treatment was replicated five times. Experimental procedures and analytical methods followed those described in Section 2.3.1.

#### 2.3.5. Effect of Temperature on Mycelial Growth

Using the comprehensive PDA medium as the basal medium, mycelial cultures were incubated at five temperatures (10 °C, 15 °C, 20 °C, 25 °C, and 30 °C) in a constant-temperature incubator. Each treatment was conducted with five replicates. Experimental procedures and analytical methods were consistent with those described in Section 2.3.1.

#### 2.3.6. Orthogonal Experimental Design

Based on the results of the single-factor experiments, carbon source, nitrogen source, and inorganic salt were selected as the three experimental factors. An orthogonal design with three factors at three levels was constructed using the L_9_(3^3^) orthogonal array (Table 1). Experimental procedures were performed as described in Section 2.3.1.

### 2.4. Determination of Basic Nutritional Components of Mycelia

#### 2.4.1. Determination of Crude Polysaccharide, Crude Protein, and Crude Fat Contents

##### Crude Polysaccharide Content

The crude polysaccharide content of *G. borealis* mycelia was determined using the phenol–sulfuric acid spectrophotometric method as described in NY/T 1676–2023 [12], with slight modifications. Briefly, dried mycelial powder was extracted with hot water, and the extract was precipitated with ethanol. The precipitate was redissolved in distilled water and reacted with phenol and concentrated sulfuric acid. The absorbance was measured at 490 nm using a UV–Vis spectrophotometer(LJ–UV90A, Shandong Lanjing Electronic Technology Co., Ltd., Shandong, China), and the polysaccharide content was calculated against a glucose standard curve.

##### Crude Protein Content

The crude protein content was determined by the Kjeldahl method according to GB 5009.5–2016 [13], with slight modifications. Dried mycelial samples were digested with concentrated sulfuric acid in the presence of a catalyst, followed by distillation with sodium hydroxide. The liberated ammonia was absorbed in a boric acid solution and titrated with standard hydrochloric acid. The protein content was calculated by multiplying the nitrogen content by a conversion factor of 6.25.

##### Crude Fat Content

The crude fat content was determined using the Soxhlet extraction method (the first method in GB 5009.6–2016) [14], with slight modifications. Dried mycelial powder was extracted with anhydrous petroleum ether in a Soxhlet apparatus for 6–8 h. The solvent was then evaporated, and the residue was dried to constant weight at 105 °C. The crude fat content was calculated as the percentage of the extracted fat of the original sample weight.

#### 2.4.2. Determination of Amino Acid Content

The amino acid composition of *G. borealis* mycelia was analyzed using an automatic amino acid analyzer according to GB 5009.124–2016 [15], with slight modifications. Dried mycelial samples were hydrolyzed with 6 mol/L hydrochloric acid under vacuum at 110 °C for 22 h. After hydrolysis, the acid was removed by evaporation, and the residue was dissolved in sodium citrate buffer (pH 2.2). The amino acids were separated by cation-exchange chromatography and detected by post-column ninhydrin derivatization, with identification and quantification performed by comparison with amino acid standard solutions. Tryptophan was not determined due to its destruction during acid hydrolysis.

#### 2.4.3. Determination of Trace Elements and Heavy Metals

The contents of trace elements (Fe, Zn) and heavy metals (As, Cd, Hg, Pb, Tl) in *G. borealis* mycelia were determined by inductively coupled plasma mass spectrometry (ICP–MS) using the first method described in GB 5009.268–2016 [16], with slight modifications. Dried mycelial samples were digested with concentrated nitric acid in a microwave digestion system. After cooling, the digested solution was diluted to a fixed volume with ultrapure water and filtered through a 0.45 μm membrane. The concentrations of elements were determined using an ICP–MS instrument equipped with a collision/reaction cell to eliminate polyatomic interferences. Quantification was performed by external calibration with multi-element standard solutions, and the results were expressed as mg/kg dry weight.

## 3. Results

### 3.1. Morphological and ITS-Based Identification

**Figure 1:**
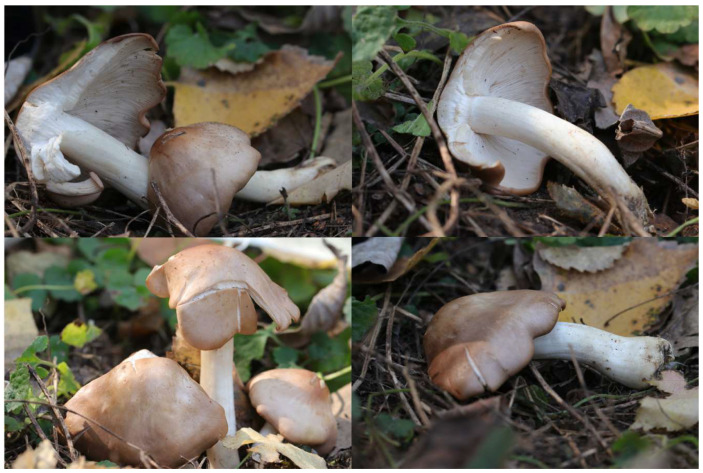
*Gerhardtia borealis*: Basidiomata.

The pileus of strain HMJU10471 measured 25–68 mm in diameter, hemispherical, with an undulate margin. The surface was smooth, glabrous, dull, dry, and non-hygrophanous, ranging in color from yellowish-brown to light yellowish-brown, with a darker center. The lamellae were adnate, crowded, with smooth edges, and creamy white in color. The stipe measured 5.2–6.3 mm in length and 7–9 mm in width; it was cylindrical, equal in width, occasionally slightly curved, and creamy white to pale yellowish-brown. The basal surface was covered with silky fibrils and remained dry. The context was white (Figure 1).

A BLAST analysis of strain HMJU10471 against the NCBI database showed that it shared 100.00% sequence identity with *G. borealis*, with the GenBank accession number MW724270.1. Combined with the morphological observations, strain HMJU10471 was conclusively identified as *G. borealis*.

### 3.2. Biological Characteristics Analysis

#### 3.2.1. Effect of pH on Mycelial Growth

**Figure 2:**
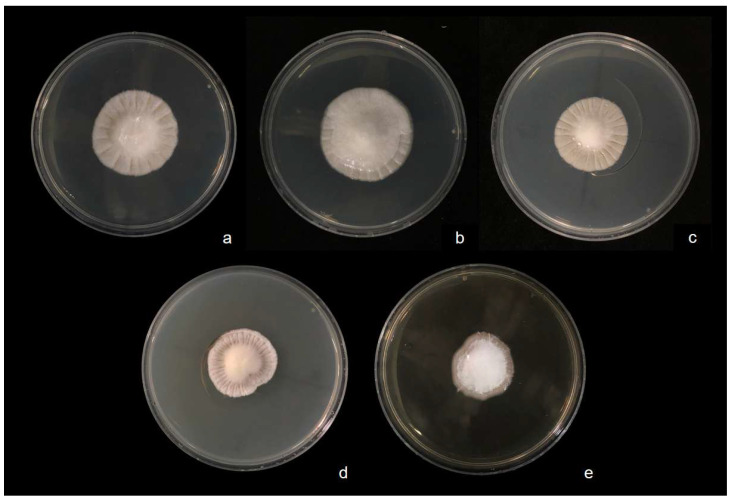
Effects of different pH on mycelial growth of *G. borealis.* Representative images of mycelial growth at various pH levels: (**a**) pH5.0; (**b**) pH6.0; (**c**) pH7.0; (**d**) pH8.0; (**e**) pH9.0.

As shown in Figure 2 and Table 2, strain HMJU10471 was able to grow on the comprehensive PDA medium across a pH range of 5.0–9.0. However, significant differences in mycelial growth rate were observed among treatments. The growth rates ranked as follows: pH 6.0 > pH 5.0 > pH 7.0 > pH 8.0 > pH 9.0. The highest growth rate was recorded at pH 6.0 (0.939 ± 0.021 mm/d), which was significantly higher than those at pH 7.0, 8.0, and 9.0. Although the growth rate at pH 5.0 exceeded that at pH 7.0, the difference was not statistically significant. In terms of growth vigor, *G*. *borealis* exhibited robust and dense mycelial growth within the pH range of 5.0–6.0, with optimal vigor observed at pH 6.0. In conclusion, the optimal pH for mycelial growth of strain HMJU10471 was determined to be 6.0.

#### 3.2.2. Effect of Carbon Sources on Mycelial Growth

**Figure 3:**
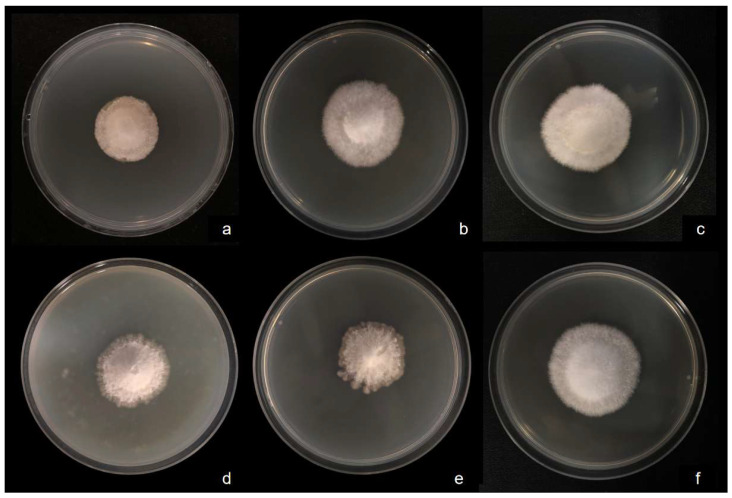
Effects of different carbon sources on mycelial growth of *G. borealis.* Representative images of mycelial growth in various media: (**a**) CK; (**b**) glucose; (**c**) sucrose; (**d**) fructose; (**e**) starch; (**f**) lactose.

As shown in Figure 3, strain HMJU10471 was able to grow on media containing all tested carbon sources. The mycelial growth rates ranked as follows: lactose > sucrose > glucose > CK > fructose > starch. The highest growth rates were observed on media supplemented with lactose and sucrose, reaching 1.171 mm/d and 1.116 mm/d, respectively (Table 3), with dense and vigorous mycelial growth. Growth on glucose was slightly lower (1.084 mm/d), ranking third. In contrast, the slowest growth occurred on starch-containing medium (0.928 mm/d), where the mycelia were sparse and exhibited weak growth vigor. No significant differences were observed among glucose, sucrose, and lactose treatments. Based on a comprehensive evaluation of growth rate and mycelial vigor, lactose was identified as the optimal carbon source for mycelial growth of *G*. *borealis*.

#### 3.2.3. Effect of Nitrogen Sources on Mycelial Growth

**Figure 4:**
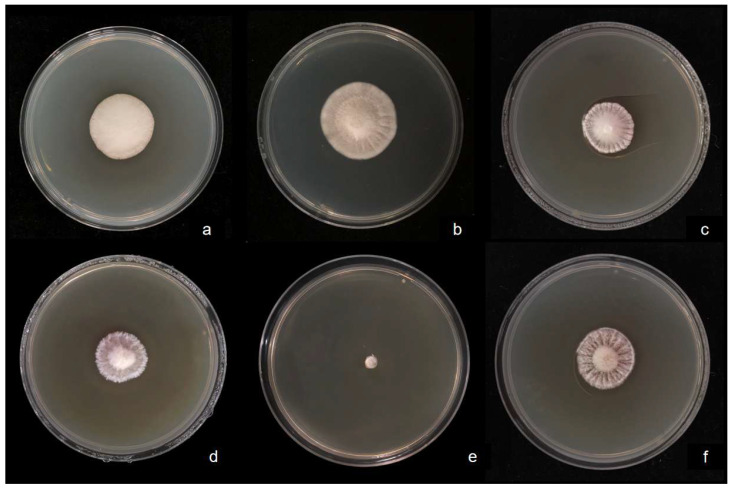
Effects of different nitrogen sources on mycelial growth of *G. borealis.* Representative images of mycelial growth in various media: (**a**) CK; (**b**) NH_4_NO_3_; (**c**) beef powder peptone; (**d**) yeast powder; (**e**) urea; (**f**) KNO_3_.

As shown in Figure 4 and Table 4, *G*. *borealis* was able to grow on media containing all tested nitrogen sources except urea. The mycelial growth rates follow: NH_4_NO_3_ > CK > KNO_3_ > peptone > beef extract > urea. The highest growth rate was observed on medium supplemented with NH_4_NO_3_ (1.070 mm/d), where the mycelia were dense and exhibited strong growth vigor. No mycelial growth was detected when urea was used as the sole nitrogen source. Based on a comprehensive assessment of growth rate and mycelial vigor, NH_4_NO_3_ was identified as the optimal nitrogen source for mycelial growth of *G. borealis*.

#### 3.2.4. Effect of Inorganic Salts on Mycelial Growth

**Figure 5:**
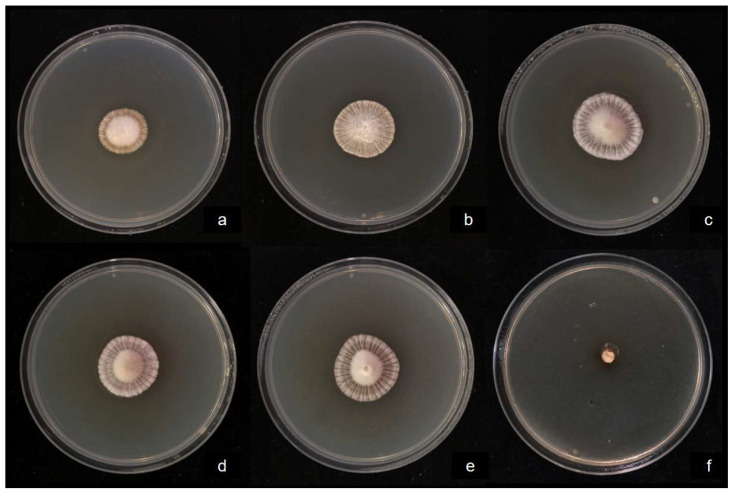
Effects of different inorganic salts on mycelial growth of *G. borealis.* Representative images of mycelial growth in various media: (**a**) CK; (**b**) MgSO_4_; (**c**) (NH_4_)_2_SO_4_; (**d**) KCl; (**e**) NaCl; (**f**) Fe_2_(SO_4_)_3_.

As shown in Figure 5 and Table 5, *G*. *borealis* was able to grow on media containing all tested inorganic salts except Fe_2_(SO_4_)_3_. The mycelial growth rates ranked as follows: NaCl > MgCl_2_ > KCl > (NH_4_)_2_SO_4_ > CK > Fe_2_(SO_4_)_3_. The highest growth rate was observed on medium supplemented with NaCl (0.926 mm/d), where the mycelia were dense and exhibited strong growth vigor. In contrast, no mycelial growth was detected when Fe_2_(SO_4_)_3_ was used. Based on a comprehensive evaluation of growth rate and mycelial vigor, NaCl was identified as the optimal inorganic salt for mycelial growth of *G. borealis*.

#### 3.2.5. Effect of Temperature on Mycelial Growth

**Figure 6:**
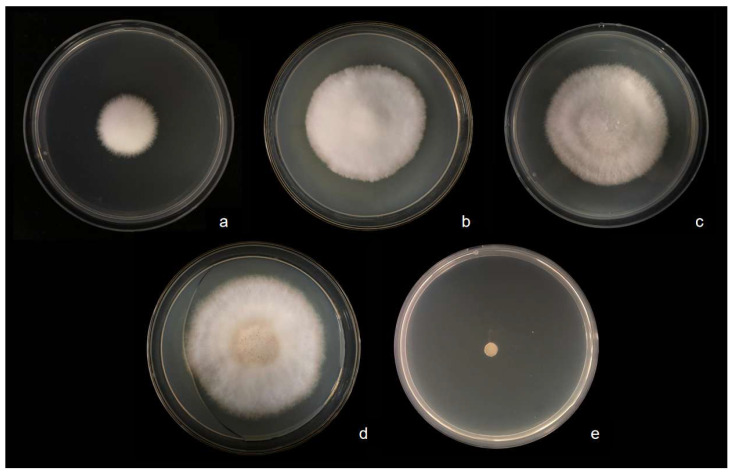
Effects of different temperatures on mycelial growth of *G. borealis.* Representative images of mycelial growth at different temperatures: (**a**) 10 °C; (**b**) 15 °C; (**c**) 20 °C; (**d**) 25 °C; (**e**) 30 °C.

As shown in Figure 6 and Table 6, *G*. *borealis* exhibited mycelial growth within the temperature range of 10–25 °C, whereas no growth was observed at 30 °C. The growth rates ranked as follows: 25 °C > 20 °C > 15 °C > 10 °C > 30 °C. The highest growth rate was recorded at 25 °C (1.027 mm/d), with dense and vigorous mycelia, showing extremely significant differences compared with other temperature treatments. At 20 °C, the growth rate (0.836 mm/d) was slower than that at 25 °C; however, the mycelia remained dense and vigorous, with no significant difference compared to those at 15 °C. No mycelial growth was detected at 30 °C. Based on a comprehensive evaluation of growth rate and mycelial vigor, the optimal temperature for mycelial growth of *G. borealis* was determined to be 25 °C.

#### 3.2.6. Results of the Orthogonal Experiment

Based on the results of single-factor tests, three factors that greatly influenced mycelial growth were selected: nitrogen source (A), carbon source (B), and inorganic salt (C). An L_9_(3^3^) orthogonal design was adopted to carry out a three-factor, three-level experiment (Table 1), with mycelial growth rate as the response indicator. The results are shown in Table 7.

Range analysis indicated that the order of influence of the three factors on mycelial growth rate was carbon source (B) > nitrogen source (A) > inorganic salt (C), with range (R) values of 0.426, 0.392, and 0.148, respectively. The carbon source had the largest range value, suggesting that the type of carbon source is the most critical nutritional factor regulating mycelial growth in *G. borealis*. Analysis of the k values for the nitrogen source showed k_1_ (0.994) > k_3_ (0.945) > k_2_ (0.602); for the carbon source, k_2_ (1.093) > k_3_ (0.779) > k_1_ (0.668); and for the inorganic salt, k_3_ (0.897) > k_1_ (0.894) > k_2_ (0.749). Accordingly, the optimal theoretical combination was determined to be A_1_B_2_C_3_, namely ammonium nitrate as the nitrogen source, sucrose as the carbon source, and magnesium sulfate as the inorganic salt. Under this optimized combination (Combination 2), the mycelial growth rate reached 1.379 ± 0.010 mm/d, which was significantly higher than that of all other treatments except Combinations 1 and 6 (*p* < 0.01), with dense, white, and vigorously growing mycelia, confirming it as the optimal culture medium formulation.

Variance analysis of the orthogonal experiment (Table 8) further validated the conclusions of the range analysis. The carbon source exhibited the highest mean square (0.146) and F value (4.368), followed by the nitrogen source (mean square 0.137, F = 4.089), and the inorganic salt was the lowest (mean square 0.022, F = 0.646). The order of influence of the three factors on mycelial growth rate was consistent with that from the range analysis. Notably, although the *p* values for each factor did not reach the 0.05 significance level (*p* = 0.186–0.608), this may be attributable to the low degrees of freedom (df = 2) of the orthogonal design, leading to insufficient test power. Nevertheless, the F values clearly reflected the relative importance of each factor, with the effect sizes of carbon source and nitrogen source being much larger than that of inorganic salt, suggesting that precise regulation of carbon and nitrogen nutrition should be prioritized in subsequent domestication and cultivation efforts.

A comprehensive comparison of the single-factor and orthogonal experiment results revealed that, although lactose was the best carbon source in single-factor experiments, the combination with sucrose (A_1_B_2_C_3_) under the multi-factor interaction of the orthogonal design achieved superior growth performance. This phenomenon highlights the complex interactive effects among different nutritional components, indicating that the results of single-factor optimization cannot be simply extrapolated to a complex culture medium system. It further confirms the necessity of using orthogonal design or multi-factor response surface methodology for comprehensive optimization.

**Figure 7:**
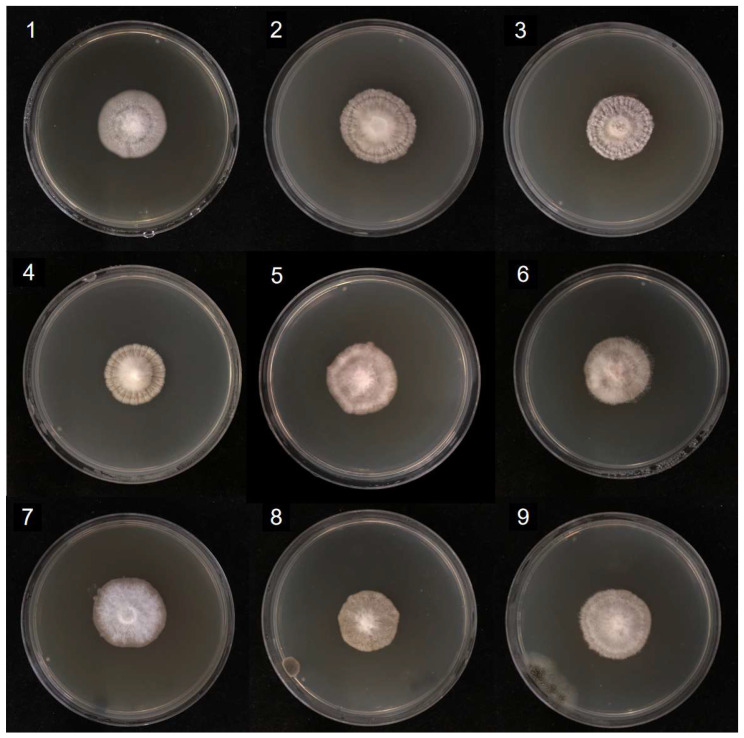
Mycelial growth of *Gerhardtia borealis* in the nine orthogonal test combinations (1–9) listed in Table 7. Each number corresponds to a specific combination of nitrogen source, carbon source, and inorganic salt: (1) no nitrogen, glucose, NaCl; (2) NH_4_NO_3_, sucrose, MgSO_4_; (3) no nitrogen, lactose, MgSO_4_; (4) NH_4_NO_3_, glucose, KCl; (5) KNO_3_, glucose, MgSO_4_; (6) no nitrogen, sucrose, KCl; (7) KNO_3_, sucrose, NaCl; (8) KNO_3_, lactose, KCl; (9) NH_4_NO_3_, lactose, NaCl. The mycelial growth rate and vigor for each combination are presented in Table 7.

Based on the above analysis, the optimal mother culture medium formulation for mycelial growth of *G. borealis* was determined as follows: sucrose 20 g/L, ammonium nitrate 2 g/L, and magnesium sulfate 2 g/L. Under this formulation, the mycelial growth rate reached 1.379 mm/d, which was significantly better than that of the basal comprehensive PDA medium and other orthogonal combinations, providing key technical parameters for subsequent liquid spawn preparation and artificial domestication cultivation.The mycelial colony morphologies of the nine orthogonal test combinations are presented in Figure 7.

### 3.3. Analysis of Basic Nutritional Components of Mycelia

The contents of crude protein, crude fat, and crude polysaccharide in *G*. *borealis* mycelia are shown in Table 9. The results showed that the crude protein content reached 26.01%, which is higher than that reported for most edible mushrooms. The crude fat content was 6.03%, representing a moderate-to-relatively high level among edible fungi, whereas the crude polysaccharide content was comparatively low at 1.24%.

Essential amino acids accounted for 7.93 g/kg (30.39%) of the total, whereas non-essential amino acids comprised 18.16 g/kg (69.61%). Among the individual amino acids, arginine was the most abundant (5.50 g/kg, 21.08% of total amino acids), followed by alanine (4.33 g/kg, 16.60%). In contrast, aspartic acid and cystine were present at relatively low levels, at 0.18 g/kg (0.69%) and 0.08 g/kg (0.31%), respectively(Table 10).

### 3.4. Analysis of Trace Elements and Heavy Metals

The contents of trace elements and heavy metals in *G*. *borealis* mycelia are shown in Table 11. On a per-kilogram basis, the concentrations of essential elements were 28.09 mg/kg for iron and 22.17 mg/kg for zinc. The levels of potentially toxic elements were 0.06 mg/kg for arsenic, 0.13 mg/kg for cadmium, 0.01 mg/kg for mercury, and 0.19 mg/kg for lead. All measured values complied with the limits specified in GB 2762-2022 “Maximum Levels of Contaminants in Foods” [17] and the agricultural industry standard “Green Food—Edible Fungi” [18].

## 4. Discussion

Based on the biological characteristics study, the optimal temperature for *G. borealis* mycelial growth was 25 °C, with an optimal pH of 6.0. This result aligns with the characteristics of most known species within the Lyophyllaceae family. For instance, the optimal mycelial growth temperature for *Lyophyllum decastes* ranges from 20 to 25 °C, with an optimal pH of approximately 7.0 [19,20]. However, *G. borealis* exhibited a more pronounced preference for a weakly acidic environment (pH 6.0), mirroring the optimal pH of 5.0–6.0 observed in the confamilial species *Flammulina yunnanensis* [21], a preference potentially linked to its saprophytic habit and the slightly acidic soil of its native habitat. Regarding carbon source utilization, the optimal carbon source for this fungus was lactose, a finding that diverges from reports identifying maltose or glucose as the best carbon source for *Flammulina rossica* [22], suggesting potential specificity in the carbon metabolic pathways among different species. A particularly notable finding was the robust growth of this strain on the control medium devoid of any added nitrogen source. Without any nitrogen supplementation, the mycelial growth rate (1.036 mm/d) was not statistically different from that observed on the medium supplemented with ammonium nitrate, the optimal nitrogen source. This starkly contrasts with species such as *Flammulina yunnanensis*, which exhibit a strong preference for organic nitrogen sources like peptone [21]. This phenomenon may stem from the strain’s efficient utilization of endogenous nitrogen reserves within the potato-based medium or, more intriguingly, from a potential “nitrogen-saving” or enhanced nitrogen-scavenging capacity [23]. This trait holds significant biotechnological potential, warranting further investigation into its underlying enzymatic mechanisms (e.g., urease or protease activity) and the genomic basis of this response. Indeed, this finding complicates standard culture optimization approaches, indicating that the nitrogen requirements of this species are more nuanced than a simple source preference.

The results of the orthogonal experiment further challenge simplistic interpretations of nutritional optimization. We empirically determined the most effective medium combination to be ammonium nitrate, sucrose, and magnesium sulfate, which achieved a mycelial growth rate of 1.379 mm/d. Range analysis indicated that carbon and nitrogen sources were the most influential factors. Crucially, however, the analysis of variance revealed that the effects of the three tested factors—carbon source, nitrogen source, and inorganic salts—on the mycelial growth rate did not reach statistical significance at the *p* < 0.05 level. We interpret this as evidence of intricate interdependencies and interactive effects among the medium components within the tested concentration ranges. The shift in the most effective carbon source from lactose in the single-factor experiment to sucrose in the orthogonal combination is a clear empirical manifestation of these interactions, a phenomenon similarly documented in nutritional optimization studies of other edible fungi [24]. Given this, the proposed medium formulation should not be regarded as a definitive optimum but rather as an improved starting point for more sophisticated statistical optimization. For instance, response surface methodology (RSM) [25,26], which effectively resolves interactive effects among multiple factors by constructing continuous variable surface models, has been widely applied to culture medium optimization for edible fungi. Among phylogenetically close species within the Lyophyllaceae, Shan et al. [25] utilized Box–Behnken RSM to optimize the liquid spawn medium for *Lyophyllum decastes*, achieving a 7.34-fold increase in mycelial dry weight compared to the initial formulation, fully demonstrating the method’s strength in addressing complex nutrient factor interactions. Similarly, RSM has yielded favorable outcomes in optimizing multi-factor culture conditions for edible fungi within the genus Lepista [26]. Consequently, future research, building upon the empirically derived optimal formula (A1B2C3) from this experiment, should systematically optimize the concentrations of the carbon source, nitrogen source, and inorganic salts using RSM-centric continuous surface approaches to precisely pinpoint the global optimal medium formulation.

From a nutritional perspective, *G. borealis* mycelium presents a unique nutritional profile. Its high crude protein content (26.01%) aligns with the nutritional pattern reported for other Lyophyllaceae species, such as *L. cinerescens* and *L. connata* [4,5]. However, its crude fat content (6.03%) is notably higher than the characteristically low levels (typically below 2%) observed in many edible fungi, including the closely related *L. connata*, which possesses a crude fat content of only 1.22% [5]. This serves as a key distinguishing characteristic. The value of *G. borealis* mycelium may not reside in its simple classification as a low-fat food but rather in its moderate fat content, provided the fatty acid profile is favorable, rendering it a potential candidate ingredient for formulations requiring different energy densities or specific lipid compositions, rather than being merely a generic high-protein, low-fat supplement. A total of 17 amino acids were detected in the mycelium, with a total content reaching 26.09 g/kg, of which essential amino acids accounted for 30.39%. Notably, arginine exhibited the highest content at 5.50 g/kg. As a functional amino acid, arginine plays a significant role in enhancing immunity and promoting wound healing [27]. Moreover, the mycelium is rich in beneficial trace elements such as iron (28.09 mg/kg) and zinc (22.17 mg/kg), echoing reports of zinc and copper abundance in the fruiting bodies of *Lyophyllum decastes*. In terms of safety evaluation, the levels of hazardous heavy metals, including arsenic, cadmium, mercury, and lead, were all substantially below the limit requirements stipulated by both the National Food Safety Standard “Maximum Levels of Contaminants in Foods” [17] and the agricultural industry standard “Green Food—Edible Fungi” [18], indicating its excellent safety profile as a potential food ingredient.

In conclusion, the efficient mycelial growth achieved through optimized culture conditions not only advances the artificial domestication of this species but also provides a transferable technical pathway for the large-scale cultivation of other edible fungi. Overall, this approach unlocks the ecological value and application potential of the Lyophyllaceae family, thereby promoting its diversified applications in fields such as food safety, medicine and health, and environmental management.

## 5. Conclusions

Single-factor experiments on biological characteristics showed that the optimal carbon source for mycelial growth of *Gerhardtia borealis* was lactose, the optimal nitrogen source was ammonium nitrate, the optimal inorganic salt was sodium chloride, the optimal temperature was 25 °C, and the optimal pH was 6.0. Orthogonal experimental analysis indicated that the optimal culture medium formulation for *G. borealis* mycelia consisted of sucrose (20 g/L), ammonium nitrate (2 g/L), and magnesium sulfate (2 g/L) in 1 L of water. Under this formulation, the mycelial growth rate reached 1.379 mm/d, with dense and vigorous mycelial morphology. The crude protein, crude fat, and crude polysaccharide contents of *G. borealis* mycelia were 26.01%, 6.03%, and 1.24%, respectively. The total amino acid content was 26.09 g/kg, of which essential amino acids accounted for 7.93 g/kg (30.39%) and non-essential amino acids for 18.16 g/kg (69.61%). Among the detected amino acids, arginine was the most abundant (5.50 g/kg), accounting for 21.08% of the total.

## Figures and Tables

**Table 1:** Factors and levels of orthogonal tests.

Level	Factor		
	Nitrogen Source (A)	Carbon Source (B)	Inorganic Salt (C)
1	NH_4_NO_3_	Lactose	NaCl
2	KNO_3_	Sucrose	KCl
3	No nitrogen source	Glucose	MgSO_4_

**Table 2:** The effect of different pH on mycelial growth.

pH	Mycelial Growth Rate (mm/d)	Mycelium Color	Mycelium Vigor	Significance of Difference	
				0.05	0.01
5.0	0.914 ± 0.093	white	+++	ab	A
6.0	0.939 ± 0.021	white	+++	a	A
7.0	0.836 ± 0.059	white	++	b	A
8.0	0.514 ± 0.084	white	+	c	B
9.0	0.357 ± 0.078	white	+	d	C

Note: Different uppercase letters indicate significant differences (*p* < 0.01); different lowercase letters indicate significant differences (*p* < 0.05); “+++” indicates dense, robust mycelium; “++” indicates slightly denser mycelium and slightly better growth; “+” indicates sparse, poorly growing mycelium.

**Table 3:** The effect of different carbon sources on mycelial growth.

Carbon Sources	Mycelial Growth Rate (mm/d)	Mycelium Color	Mycelium Vigor	Significance of Difference	
				0.05	0.01
CK	1.063 ± 0.033	white	++	bc	ABC
Glucose	1.084 ± 0.077	white	+++	abc	AB
Sucrose	1.116 ± 0.072	white	+++	ab	AB
Fructose	1.000 ± 0.079	white	++	cd	BC
Starch	0.928 ± 0.018	white	++	d	C
Lactose	1.171 ± 0.681	white	+++	a	A

Note: Different uppercase letters indicate significant differences (*p* < 0.01); different lowercase letters indicate significant differences (*p* < 0.05); “+++” indicates dense, robust mycelium; “++” indicates slightly denser mycelium and slightly better growth.

**Table 4:** The effect of different nitrogen sources on mycelial growth.

Nitrogen Sources	Mycelial Growth Rate (mm/d)	Mycelium Color	Mycelium Vigor	Significance of Difference	
				0.05	0.01
CK	1.036 ± 0.090	white	+++	a	A
NH_4_NO_3_	1.070 ± 0.036	white	+++	a	A
beef extract	0.496 ± 0.011	white	+	c	C
peptone	0.653 ± 0.030	white	+	b	B
urea	–	–	–	d	D
KNO_3_	0.980 ± 0.060	white	++	a	A

Note: Different uppercase letters indicate significant differences (*p* < 0.01); different lowercase letters indicate significant differences (*p* < 0.05); “+++” indicates dense, robust mycelium; “++” indicates slightly denser mycelium and slightly better growth; “+” indicates sparse, poorly growing mycelium; the symbol "–" indicates no growth.

**Table 5:** The effect of different inorganic salts on mycelial growth.

Inorganic Salts	Mycelial Growth Rate (mm/d)	Mycelium Color	Mycelium Vigor	Significance of Difference	
				0.05	0.01
CK	0.686 ± 0.050	white	++	c	C
MgSO_4_	0.873 ± 0.011	white	+++	ab	A
(NH_4_)_2_SO_4_	0.753 ± 0.060	white	++	c	BC
KCl	0.836 ± 0.030	white	+++	b	AB
NaCl	0.926 ± 0.045	white	+++	a	A
Fe_2_(SO_4_)_3_	–	–	–	d	D

Note: Different uppercase letters indicate significant differences (*p* < 0.01); different lowercase letters indicate significant differences (*p* < 0.05); “+++” indicates dense, robust mycelium; “++” indicates slightly denser mycelium and slightly better growth; the symbol "–" indicates no growth.

**Table 6:** The effect of different temperatures on mycelial growth.

Temperature	Mycelial Growth Rate (mm/d)	Mycelium Color	Mycelium Vigor	Significance of Difference	
				0.05	0.01
10 °C	0.188 ± 0.034	white	+	c	C
15 °C	0.769 ± 0.071	white	++	b	B
20 °C	0.836 ± 0.031	white	+++	b	B
25 °C	1.027 ± 0.201	white	+++	a	A
30 °C	–	–	–	d	D

Note: Different uppercase letters indicate significant differences (*p* < 0.01); different lowercase letters indicate significant differences (*p* < 0.05); “+++” indicates dense, robust mycelium; “++” indicates slightly denser mycelium and slightly better growth; “+” indicates sparse, poorly growing mycelium; the symbol "–" indicates no growth.

**Table 7:** Orthogonal test result of *G. borealis* mycelial growth.

Combination	Factor			Mycelial Growth Rate (mm/d)	Mycelium Color	Mycelium Vigor
	Nitrogen Source (A)	Carbon Source (B)	Inorganic Salt (C)			
1	3 (no nitrogen)	3 (glucose)	1 (NaCl)	1.103 ± 0.061 bB	white	+++
2	1 (NH_4_NO_3_)	2 (sucrose)	3 (MgSO_4_)	1.379 ± 0.010 aA	white	+++
3	3 (no nitrogen)	1 (lactose)	3 (MgSO_4_)	0.699 ± 0.018 dCD	white	+
4	1 (NH_4_NO_3_)	3 (glucose)	2 (KCl)	0.711 ± 0.032 dCD	white	+
5	2 (KNO_3_)	3 (glucose)	3 (MgSO_4_)	0.613 ± 0.065 dDE	white	++
6	3 (no nitrogen)	2 (sucrose)	2 (KCl)	1.122 ± 0.181 bB	white	+++
7	2 (KNO_3_)	2 (sucrose)	1 (NaCl)	0.779 ± 0.132 cdCD	white	+++
8	2 (KNO_3_)	1 (lactose)	2 (KCl)	0.413 ± 0.101 eE	white	+
9	1 (NH_4_NO_3_)	1 (lactose)	1 (NaCl)	0.891 ± 0.121 cBC	white	+++
K1	2.981	2.003	2.683			
K2	1.805	3.280	2.246			
K3	2.834	2.337	2.691			
k1	0.994	0.668	0.894			
k2	0.602	1.093	0.749			
k3	0.945	0.779	0.897			
R	0.392	0.426	0.148			

Note: Different uppercase letters indicate significant differences (*p* < 0.01); different lowercase letters indicate significant differences (*p* < 0.05); “+++” indicates dense, robust mycelium; “++” indicates slightly denser mycelium and slightly better growth; “+” indicates sparse, poorly growing mycelium.

**Table 8:** Analysis of variance of orthogonal test results.

Source	Type III Sum of Squares	*df*	Mean Squares	F	*p*
Corrected Model	0.609 ^a^	6	0.102	3.034	0.269
Intercept	6.452	1	6.452	192.756	0.005
A	0.274	2	0.137	4.089	0.197
B	0.292	2	0.146	4.368	0.186
C	0.043	2	0.022	0.646	0.608
Error	0.067	2	0.033		
Total	7.128	9			
Corrected Total	0.676	8			

Note: ^a^ R^2^ = 0.901 (Corrected Model SS/Corrected Total SS).

**Table 9:** Basic nutrition ingredients of *G. borealis*.

Basic Nutritional Composition	Content
Crude protein	26.01 ± 0.04
Crude fat	6.03 ± 0.25
Crude polysaccharides	1.24 ± 0.18

**Table 10:** Composition and content of amino acids in mycelium of *G. borealis* (g/kg).

Amino Acid Name	Content
Aspartic acid	0.18 ± 0.23
Glutamic acid	3.64 ± 0.03
Serine	1.48 ± 0.04
Glycine	0.85 ± 0.06
Histidine	0.43 ± 0.11
Arginine	5.50 ± 0.08
Threonine *	0.93 ± 0.04
Alanine	4.33 ± 0.02
Proline	0.79 ± 0.05
Tyrosine	0.88 ± 0.09
Valine *	1.17 ± 0.15
Methionine *	0.33 ±0.22
Cysteine	0.08 ± 0.06
Isoleucine *	0.90 ± 0.02
Leucine *	1.97 ± 0.06
Phenylalanine *	1.36 ± 0.03
Lysine *	1.27 ± 0.05
Essential amino acid content	7.93
Non–essential amino acid content	18.16
Total	26.09

Note: * Represents essential amino acids.

**Table 11:** Contents of heavy metals and trace elements in mycelia of *G. borealis* (mg/kg).

Element Names	Content	GB 2762–2022 Limit for Contaminants in Foods (mg/kg)	NY/T 749–2023 Green Food-Edible Fungi (mg/kg)
Fe	28.09 ± 0.04		
Zn	22.17 ± 0.17		
Tl	not detected (<0.005)		
As	0.06 ± 0.01	0.5	1.0
Cd	0.13 ± 0.03	0.2	1.0
Hg	0.01 ± 0.00	0.1	0.2
Pb	0.19 ± 0.02	0.5	2.0

## Data Availability

The original contributions presented in this study are included in the article. Further inquiries can be directed to the corresponding authors.
